# Diagnostic Efficacy of Synthesized 2D Digital Breast Tomosynthesis in Multi-ethnic Malaysian Population

**DOI:** 10.1038/s41598-018-37451-4

**Published:** 2019-02-06

**Authors:** N. Ab Mumin, K. Rahmat, F. Fadzli, M. T Ramli, C. J. Westerhout, N. Ramli, F. I. Rozalli, K. H. Ng

**Affiliations:** 1 Biomedical Imaging Department, University Malaya Research Imaging Centre, Kuala Lumpur, Malaysia; 2 Radiology Department, Faculty of Medicine, University Teknologi MARA, Selangor, Malaysia

## Abstract

Synthesized 2D images can be reconstructed from tomosynthesis images in breast imaging. This study aims to investigate the diagnostic efficacy of synthesized 2D images (C-View) in comparison to full field digital mammography (FFDM) when used with digital breast tomosynthesis (DBT) in multi-ethnic Malaysian population. FFDM and C-View images (n = 380) were independently evaluated by three readers through Breast Imaging Reporting and Data System (BI-RADS) categorisation, breast density and lesion characterisation. Statistical analysis was done comparing sensitivity, specificity, positive predictive value (PPV) and negative predictive value (NPV) of C-View + DBT with FFDM + DBT as standard of reference. Very good interreader agreement in BI-RADS category and density assessment between C-View + DBT and FFDM + DBT, with high sensitivity, specificity, PPV and NPV of C-View + DBT when compared with FFDM + DBT. There was comparable PPV between C-View + DBT and FFDM + DBT, with histopathology as gold standard. High level of interreader agreement in BI-RADS category and density assessment for FFDM + DBT and C-View + DBT. There was good agreement between FFDM + DBT and C-View + DBT in mass characterization, and almost perfect agreement in calcification and asymmetric density. 52.2% lower radiation dose incurred when using C-View + DBT. Hence, synthesized 2D images are comparable to FFDM with reduction in radiation dose within the limits of Malaysian multi-ethnic population.

## Introduction

Digital breast tomosynthesis (DBT) is a relatively new technology in breast imaging that has shown great promise in solving the problems of overlapping breast tissues obscuring a pathology, or mimicking a pathology in full field digital mammography (FFDM)^1–4^. Mammography is known to be the most effective method in detecting breast cancer at an early stage and reducing breast cancer mortality. However, with FFDM, approximately 30% of cancers are missed, and in some cases, patients are subjected to unnecessary biopsy and distress^3^.

The current practice in performing DBT is the combination of the FFDM views (craniocaudal (CC) and mediolateral oblique (MLO)) with additional tomosynthesis in these views too. This is to allow comparison with previous mammographic studies and avoiding clusters of microcalcifications to be wrongly interpreted on tomosynthesis view only^5^. This combined procedure has resulted in reduced recall rates with increases in invasive cancer detection and improved diagnostic accuracy over FFDM for soft–tissue density lesions^4,6^.

Unfortunately this means increasing the radiation dose^7–9^, albeit still within the Mammography Quality Standards Acts (MQSA) recommendation which limits the average glandular dose to 3mGy per breast. Development of synthesized 2D images is an attempt to reduce the radiation dose by 45–50%, by obviating the exposure for FFDM^7,9,10^. The synthesized 2D images in this study is known as C-View, develop by Hologic, and approved by the U.S Food and Drug Administration in May 2013. It directly generates the 2D images via slab reconstruction from the 3D dataset.

Several papers have been published on either reader studies or prospective studies looking into comparability between synthesized 2D images and FFDM^7–12^. Results from three studies showed that FFDM and synthesized images are comparable^9,10,12^. Another reader study showed lower sensitivity and comparable specificity in synthesized 2D images and DBT versus FFDM and DBT. One study included symptomatic patients^9^, whilst the rest were on screening population. In conclusion, these early studies demonstrated that the synthesized mammography imaged from tomosynthesis may be an acceptable alternative to FFDM, as evidenced by reduced recall rates and comparable cancer detection rates.

However, none of these studies looked into women in an Asian population, who have higher breast densities^13–15^ and tend to develop breast cancer at a younger age (40–50 years old for Asians versus 70–80 years old in Western countries)^16^.

The purpose of our study is to assess whether the synthesized 2D images are comparable to FFDM in BI-RADS categorisation, breast density assessment, detection of calcifications, mass, asymmetric density and architectural distortions when reviewed with tomosynthesis. We hypothesized that the synthesized 2D images are equivalent to FFDM and can replace FFDM in routine clinical practice.

## Methods

### Study population

Informed consent was obtained from all included patients and ethical approval from the medical ethics committee of the University of Malaya Medical Centre. The research methodology was adherent to the institutional regulations and guidelines.

We analysed 380 women who attended the breast imaging unit at the University of Malaya Medical Centre from September 2014 to October 2015. All women were referred for breast imaging either for screening (n = 324) or presence of symptoms (n = 56). Patients with previous breast surgery, implants and known breast malignancy were excluded. Histological diagnosis was obtained in 41 patients. All the lesions which were reported as benign were followed up for two years.

### Image acquisition

All subjects underwent combined FFDM and tomosynthesis acquisition, which is the Combo mode in automatic exposure control (AEC) using Selenia Dimension machine (Hologic, Bedford, Massachusetts). The DBT X-ray tube anode used was tungsten with rhodium filter. Examinations were performed by dedicated breast imaging radiographers. During acquisition, the breast was compressed between breast plates as in conventional mammography. The x-ray tube moved in a limited arc, allowing for 11 to 15 low - dose projection images to be acquired for the tomosynthesis images. This was then followed by acquisition of 2D FFDM image prior to release of compression. Data from the low dose projection images were used to reconstruct 1-mm-thick sections separated by 1 mm space. This varied according to the thickness of the compressed breast.

### Reconstructed 2D image acquisition

Synthesized 2D images of the patients are generated from the acquired DBT images. In this study, the synthesized 2D images were produced by summing and filtering the DBT images, almost similar to maximum intensity projection method^9^. The image processing software used in this study was C-View^TM^ software by Hologic. This software is an image processing application for post processing the pixel data of DBT^17^, and does not need additional radiation exposure. A detailed explanation of the software method is described elsewhere^18^. Hence, in our study all patients will each have three sets of images i.e. conventional 2D FFDM, DBT, as well as synthesized 2D images (C-View) (see Supplementary image and video). Each set consists of CC and MLO views. The C-View images of all patients are readily generated and reproducible upon availability of DBT images.

### Independent reader assessment

The images were interpreted either from the SecurView DX workstation (Hologic Inc, Bedford, MA, USA) or accessed through electronic medical records (Picture Archiving and Communication system; RIS-I 5.0, GE Centricity) through Apple Thunderbolt Display 2560 × 1440 pixel resolution monitor. There were three readers; MR (R1), CJW (R2) and FF (R3) with each having a minimum of six years of mammographic reading experience. Assessments of FFDM and C-View images were done retrospectively by each reader in separate seating by filling in a proforma data collection sheet. The images were reviewed together with tomosynthesis images. Assessment criteria included Breast Imaging Reporting and Data System (BI-RADS), density assessment, calcifications, mass, asymmetric density and architectural distortion. If the subjects had other imaging modalities, for example breast ultrasound or magnetic resonance imaging, they were not provided to the readers. Readers were blinded to the outcome status of each case and read the cases independently of all other readers. The data obtained were collated and analysed by the first author, which was not one of the readers. Each reader had undergone training provided by Hologic prior to the commencement of study.

BI-RADS categories were scored 1 to 5; category 1 stands for no suspicion of malignancy, category 2 for benign findings, category 3 for likely benign lesions which require closer follow up, category 4 for suspicious lesions warranting histological confirmation and category 5 for suspicious masses with an appearance of cancer with recommendation of biopsy to confirm diagnosis. Category 6 cases - confirmed malignancy - were excluded from our study.

The classification for breast density was as follows: almost entirely fatty (A), scattered fibroglandular densities (B), heterogeneously dense (C), or extremely dense (D).

Calcifications were classified based on distribution and shape. Lesions were classified by shape and margin. The presence of asymmetric density and architectural distortion were also considered in the BI-RADS category assessment score.

### Statistical analysis

All statistical analyses in this study utilised SPSS 21 (IBM SPSS Statistical software), and all statistical tests with p values less than 0.05 were considered to indicate statistical significance.

Sensitivity, specificity, positive predictive value (PPV) and negative predictive value (NPV) of C-View were calculated, taking FFDM as the standard for reference. We compared likely benign (BI-RADS 1, 2 and 3) and likely malignant (BI-RADS 4 and 5) ratios between C-View and tomosynthesis (C-View + DBT) and FFDM with tomosynthesis (FFDM + DBT) images and correlated the likely malignant category to histopathology findings. Sensitivity, specificity, PPV and NPV for both C-View + DBT and FFDM + DBT were calculated in 40 subjects using histopathology as the gold standard. Cohen kappa tests (κ) were used to compare BI-RADS categories and breast density between C-View + DBT and FFDM + DBT in each reader separately. Interreader correlation coefficient was calculated to ascertain agreement between readers in BI-RADS category assessment and breast density.

Lesion characteristics, namely shape and margin of mass, calcifications, asymmetric density and architectural distortion were compared between C-View + DBT and FFDM + DBT and between readers. Kappa test was also performed to ascertain agreement in lesion characterisation.

## Results

The majority of the patients included in this study were aged between 50 and 69 (mean age: 58), with almost half being Chinese in ethnicity. There were 42 subjects with almost fatty breasts (11.1%); 130 with scattered fibroglandular density (33.9%); 154 with heterogenously dense (40.5%) and 54 with extremely dense breast pattern (14.2%). 55% of patients were in the BI-RADS density category C and D, indicating denser breasts in the population of this study. Patient characteristics and details are tabulated as follows (Table 1).

**Table 1:** Patient age and ethnicity.

Age (y)	
<40	4 (1.1%)
40–49	64 (16.8%)
50–59	147 (38.7%)
60–69	124 (32.6%)
>69	41 (10.8%)
**Ethnicity**	
Chinese	177 (46.6%)
Malay	115 (30.5%)
Indian	70 (18.4%)
Other	17 (4.5%)

Of the 380 patients included, 41 patients were subjected to biopsy and 61% (n: 25) of these were of malignant histopathology (Table 2). The majority of the reported findings were calcifications, followed by mass, asymmetric density and architectural distortion (Table 3). All the lesions which were reported as benign showed no interval cancers in the 2 years follow up imaging.

**Table 2:** Histological findings and mass sizes.

Histopathology findings (n: 41)^a^	
• Invasive ductal carcinoma	20 (48.7%)
• Ductal carcinoma *in situ*	4 (9.8%)
• Mucinous carcinoma	1 (2.4%)
• Benign (sclerosing adenosis, fibroadenoma, fibrocystic, ductal hyperplasia, inflammatory)	15 (36.5%)
• Inconclusive	1 (2.4%)*
**Size of masses with suspicious features (n: 15)** ^**b**^	
• <2.0 cm	6 (40%)
• −2.1–4.0 cm	3 (20%)
• >4.0 cm	6 (40%)

^a^Percentage calculated with denominator number of biopsies (n: 41).

^b^Percentage calculated with denominator number of masses with suspicious features (n: 15).

*Excluded from statistical analysis.

**Table 3:** Mammographic findings.

Mammographic findings^c^	(Reader 1)	(Reader 2)	(Reader 3)
• Mass/nodules	93 (24.7%)	93 (24.7%)	93 (24.7%)
Malignant features^d^	15 (16.1%)	15 (16.1%)	15 (16.1%)
Benign features^d^	78 (83.8%)	78 (83.8%)	78 (83.8%)
• Calcification	251 (66.1%)	264 (69.5%)	259 (66.1%)
Malignant features^e^	7 (2.8%)	13 (4.9%)	9 (3.5%)
Benign features^e^	244 (97.2%)	251 (95.1%)	250 (96.5%)
• Asymmetric density	5 (1.3%)	9 (2.3%)	8 (2.1%)
• Architectural distortion	14 (3.7%)	11 (2.9%)	7 (1.8%)

^c^Percentage calculated with denominator number of patients (n: 380).

^d^Percentage calculated with denominator number of masses detected (n: 93).

^e^Percentage calculated with denominator number of calcifications detected (n: 294).

All readers showed >94% sensitivity and >98% specificity with >88% PPV and >99.4% NPV of C-View + DBT when taking FFDM + DBT as gold standard and excellent agreement was noted between FFDM + DBT and C-View + DBT in all readers (κ value: 0.811, 0.888, and 0.934 p < 0.001) in BI-RADS categorisation (Table 4).

**Table 4:** Kappa value indicating agreement in each reader between FFDM + DBT and C-View + DBT and **s**ensitivity, specificity, PPV and NPV for C-View, taking FFDM as gold standard (p value < 0.001).

	READER 1	READER 2	READER 3
Kappa value	0.811	0.888	0.934
Sensitivity	94.9%	97.1%	94.3%
Specificity	98.5%	100%	99.7%
PPV	88.1%	100%	97.1%
NPV	99.4%	99.7%	99.4%

Histopathological examinations were carried out on cases reported as BI-RADS 4 and 5. There were also BI-RADS 3 cases biopsied when either the primary team or patient requested histopathological diagnosis, and when lesions were only detected on ultrasound.

When taking histopathological findings (n = 40) as gold standard, there is comparable PPV between C-View + DBT vs FFDM + DBT for R1, R2 and R3, with PPV values of 86.2%, 83.3%, and 92.6% respectively.

Agreement between readers for BI-RADS category and density assessment were tested using a two-way mixed model of interclass correlation co-efficient. There was a high level of agreement between all readers in BI-RADS category for FFDM + DBT, with an average ICC of 0.911 (95% CI 0.894–0.925, p < 0.001). A high level of agreement was also seen between all readers in BI-RADS category for C-View + DBT, with an average ICC of 0.898 (95% CI 0.879–0.915, p < 0.001).

There was a high level of agreement between all readers in density assessment for FFDM + DBT, with an average ICC of 0.898 (95% CI 0.877–0.915, p < 0.001). There was a similar level of agreement seen with density assessment for C-View + DBT between all readers; an average ICC of 0.905 (95% CI 0.886–0.921, p < 0.001).

### Mass

For specific mass lesion characterization, there was substantial concordance in the description of mass detected on both FFDM + DBT versus C-View + DBT in all readers as summarized in Table 5. There was almost perfect agreement between FFDM + DBT and C-View + DBT in all readers in mass characterization for shape of mass (κ: 0.94, 0.94, 0.81 (R1, R2, R3), p < 0.001) and for margin of mass (κ: 0.92, 0.88, 0.54, (R1, R2, R3) p < 0.001). Inter-reader agreement ranged from fair to moderate with κ: 0.39–0.505, p value < 0.001 for shape and κ: 0.36–0.68, p < 0.001 for margin.

**Table 5:** Shape, margin and density of masses in percentages (number).

Shape	Reader 1 (n:93)		Reader 2 (n:93)		Reader 3 (n:93)	
	FFDM	C-VIEW	FFDM	C-VIEW	FFDM	C-VIEW
• Irregular	13.2% (12)	13.2% (12)	19.4% (18)	19.4% (18)	8.6% (8)	8.6% (8)
• Ovoid	26.4% (24)	26.4% (24)	23.7% (22)	20.4% (19)	21.5% (20)	21.5% (20)
• Round	41.8% (38)	39.8% (37)	36.6% (34)	35.5% (33)	35.5% (33)	35.5% (33)
• Not specified	19.4% (18)	19.4% (18)	20.4% (19)	19.4% (18)	34.3% (32)	34.3% (32)
**Margin**						
• Circumscribed	83.9% (78)	80.6% (75)	66.7% (62)	61.3% (57)	67.7% (63)	64.5% (60)
• Indistinct	3.2% (3)	4.3% (4)	15.1% (14)	12.9% (12)	5.4% (5)	5.4% (5)
• Lobulated	1.1% (1)	2.2% (2)	2.2% (2)	2.2% (2)	7.5%(7)	7.5% (7)
• Spiculated	7.5% (7)	7.5% (7)	14% (13)	16.1% (15)	11.8% (11)	11.8% (11)
• Not specified	4.3% (4)	5.4% (5)	2.2% (2)	7.5% (7)	7.5% (7)	7.5% (7)

**Figure 1:**
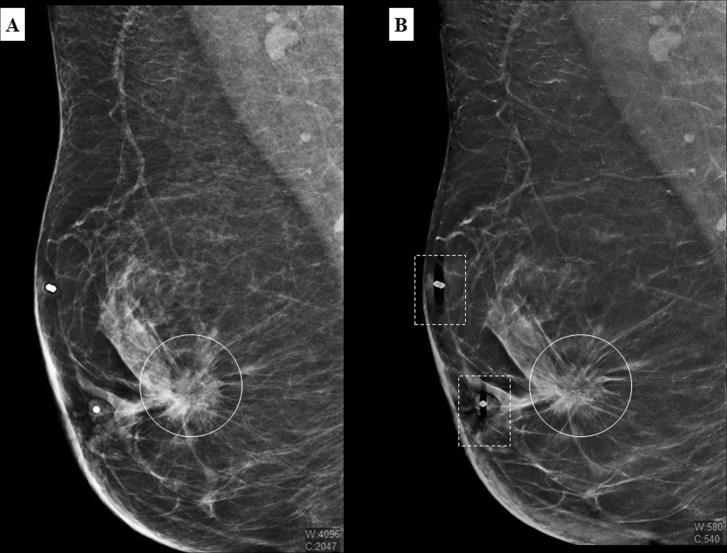
A 51 year old Malay woman who presented with a palpable lump in the right breast. MLO view of the right breast in FFDM (**A**) and C-View (**B**) with a spiculated high density lesion (white circle) detected at retroareolar region. There are artefacts produced by breast marker in C-View images (dashed square box).

**Figure 2:**
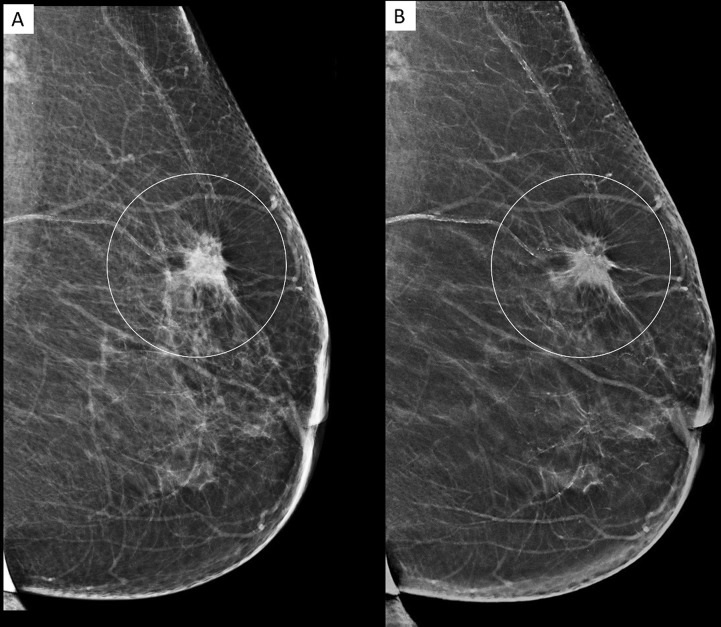
A 80 year old female who presented with a palpable lump in the left breast. MLO view of the left breast in FFDM (**A**) and C-View (**B**), showing a spiculated high density lesion (white circle) in the upper quadrant. The spiculations in C-View image are more pronounced. (BI-RADS 5). Histopathology confirmed invasive ductal carcinoma.

In conclusion, there was good agreement between FFDM and C-View in mass detection and characterization, as per example in Fig. 1. However, readers commented that spiculations in some suspicious lesions were more pronounced in the C-View image (Fig. 2).

### Calcification

The number of calcifications detected for FFDM + DBT vs C-View + DBT are; 207 vs 251 (R1), 250 vs 264 (R2), and 245 vs 259 (R3).

The percentages of calcifications detected which were classified as suspicious were 7 (2.8%), 13 (4.9%) and 9 (3.5%) for both FFDM + DBT and C-View + DBT in R1, R2 and R3 respectively. Calcifications with benign features were 200 (79.7%) vs 244 (97.2%), 237 (89.8%) vs 251 (95.1%), 236 (91.1%) vs 250 (96.5%) for FFDM + DBT vs C-View + DBT in R1, R2 and R3 respectively.

There was almost perfect agreement (κ: 0.989, 0.980, 0.894 (R1, R2, R3), p < 0.001) between FFDM + DBT and C-View + DBT for each reader in calcification detection. Inter-reader agreement yielded substantial agreement in FFDM + DBT and C-View + DBT (κ: 0.618–0.744, p < 0.001) (Table 6).

**Table 6:** Kappa value indicating inter-reader agreement in calcification in FFDM + DBT and C-View + DBT.

Interreader agreement	Kappa value	p value
Reader 1 vs Reader 2 (FFDM)	0.618	<0.001
Reader 1 vs Reader 3 (FFDM)	0.713	<0.001
Reader 2 vs Reader 3 (FFDM)	0.742	<0.001
Reader 1 vs Reader 2 (C-View)	0.618	<0.001
Reader 1 vs Reader 3 (C-View)	0.683	<0.001
Reader 2 vs Reader 3 (C-View)	0.744	<0.001

**Figure 3:**
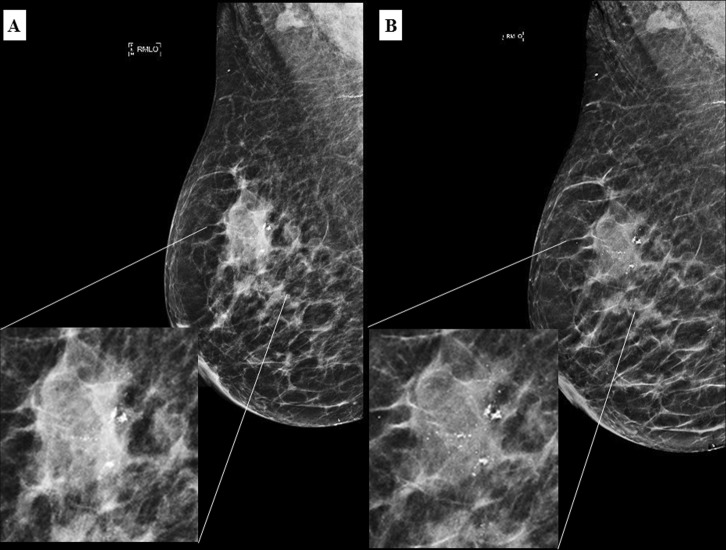
A 62 year old Malay female presented with a palpable lump in the right breast. Irregular high density mass with suspicious cluster of microcalcifications in the right upper quadrant in FFDM (**A**) and C-View (**B**) with respective magnified images. Calcifications are better seen in C-View.

**Figure 4:**
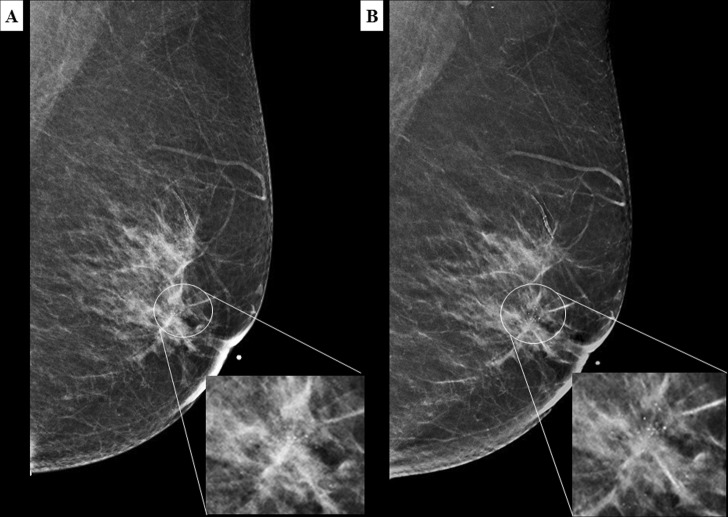
A 67 year old Chinese woman who presented with left nipple discharge. MLO view of the left breast in FFDM (**A**) and C-View (**B**) with its corresponding magnified image showing a suspicious cluster of microcalcifications in the retroareolar region (white circle). Again, the microcalcifications are clearer in C-View image. (BI-RADS 5). Histopathology confirmed a low grade ductal carcinoma *in situ*.

In some cases (Figs 3 and 4), calcifications were better seen in C-View + DBT compared to FFDM + DBT. Hence, readers reported more calcifications on C-View + DBT than FFDM + DBT, with all cases belonging to a likely benign category (BI-RADS 1, 2 and 3). There was no difference in the number of suspicious calcifications reported between C-View + DBT and FFDM + DBT in all readers.

### Asymmetric density

There were 4 (1.0%) vs 5 (1.3%) for R1, both 9 (2.3%) for R2 and both 8 (2.1%) in FFDM + DBT vs C-View + DBT for R3 for cases of asymmetric density.

There was almost perfect agreement between FFDM + DBT and C-View + DBT in all readers in asymmetric density (κ: 0.978, 0.980, 0.989 (R1, R2, R3), p < 0.05). Weighted kappa performed to test inter-reader agreement in asymmetric density showed two of the readers had substantial agreement in both FFDM + DBT and C-View + DBT (κ: 0.75, p < 0.05). However, majority of the test results were not statistically significant.

### Architectural distortion

There were 9 (2.4%) vs 13 (3.4%) for R1, 11 (2.9%) vs 15 (3.9%) for R2 and both 7 (1.8%) for R3 in FFDM + DBT vs C-View + DBT for cases of architectural distortion.

Kappa test and inter-reader agreement was computed to assess agreement between FFDM + DBT and C-View + DBT in architectural distortion, however the test results were not statistically significant.

**Figure 5:**
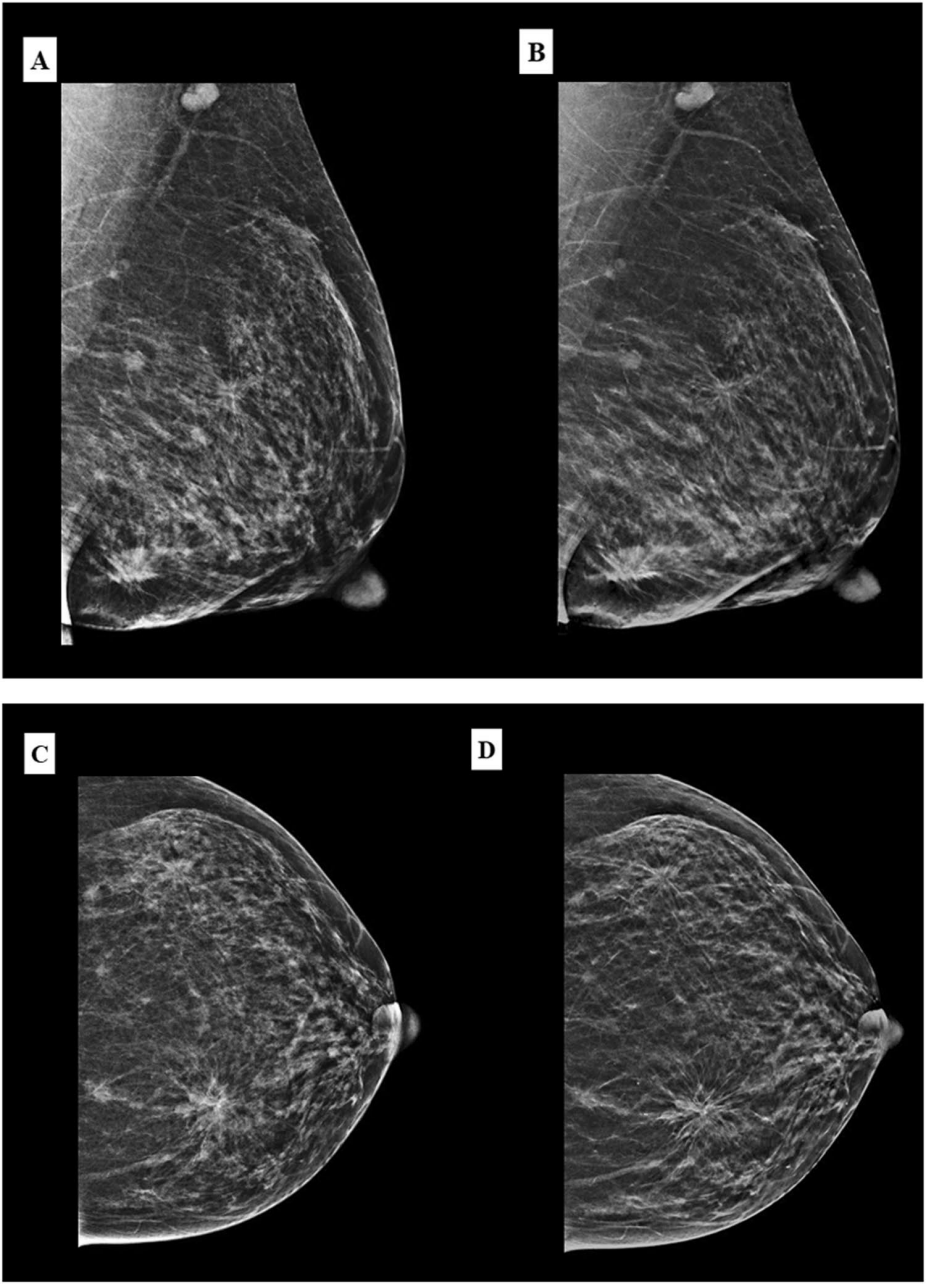
A 49 year old Malay woman who presented with palpable lumps in the left breast. MLO view of the left breast in FFDM (**A**) and C-View (**B**), and CC view in FFDM (**C**) and C-View (**D**) showing architectural distortions in both upper outer and lower inner quadrants (BI-RADS 5). The architectural distortion and spiculations are better delineated on the C-view images. Histopathology confirmed multicentric invasive ductal carcinoma.

**Figure 6:**
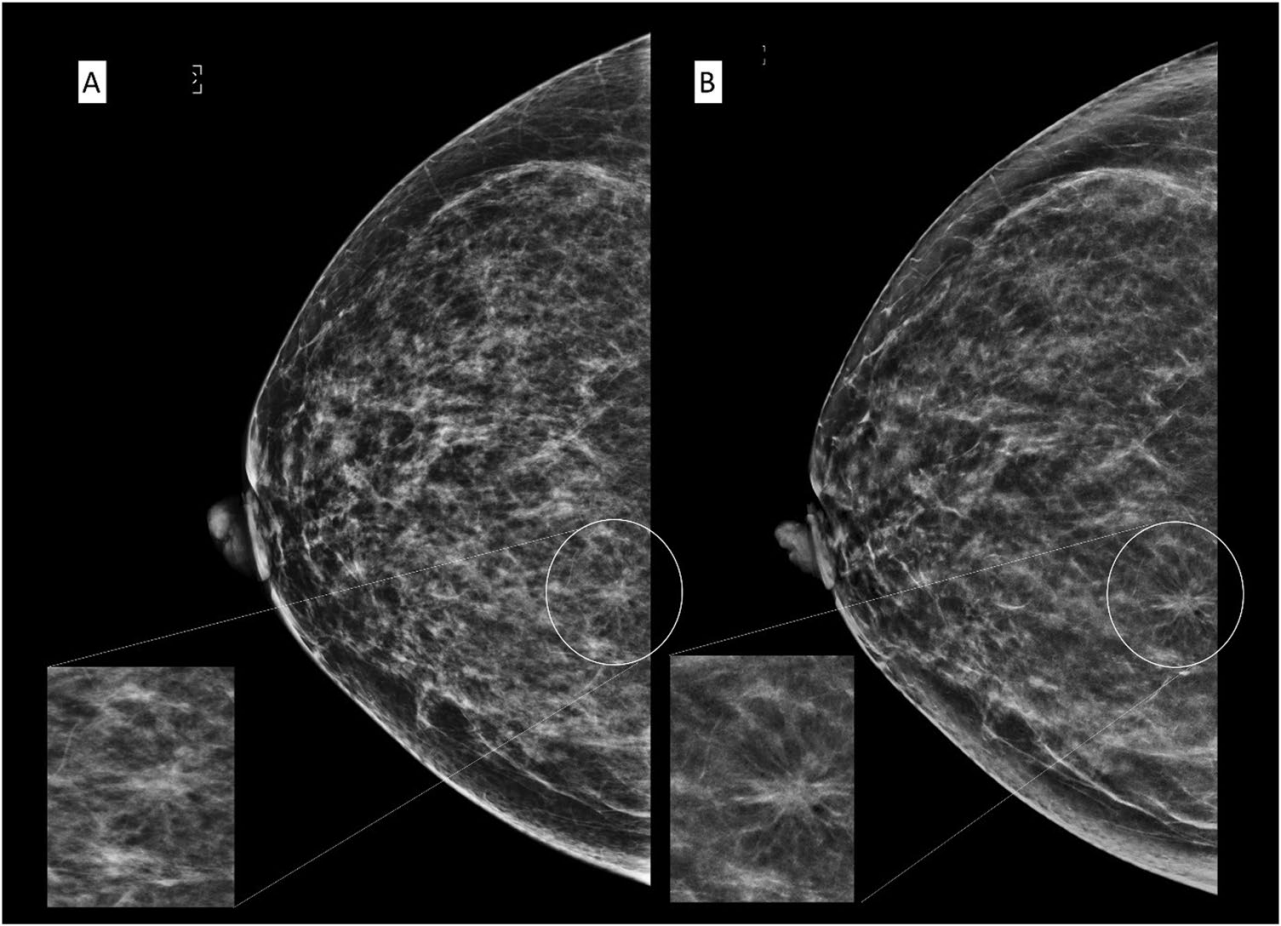
A 52 year old Chinese woman under breast surgery follow up for fibroadenoma and cysts. CC view of the right breast in FFDM (**A**) and C-View (**B**) with its magnified image showing architectural distortion in the right inner quadrant (white circle). The distortion is much better appreciated in C-View. (BI-RADS 4). Histopathology confirmed sclerosing adenosis and the lesion was excised with hook wire localisation.

Architectural distortions were better depicted in C-View images compared to FFDM as shown in Figs 5 and 6. Hence, there was more architectural distortion reported in C-View. All of the cases only seen in C-View were those with other suspicious findings (mass or calcifications). Hence, there was no significant difference in BI-RADS categorization between C-View + DBT and FFDM + DBT.

### Radiation dose

Data from 370 patients were included for radiation dose and breast compression thickness analysis. The other 10 patients were excluded as there were missing data. The average glandular dose (AGD) for FFDM plus tomosynthesis versus tomosynthesis only was 4.12mGy (95% CI 3.98–4.25) vs 1.97mGy (95% CI 1.91–2.02). In the Combo mode, the average glandular dose from FFDM contributed 52.18% of the total radiation received. Hence, by obviating FFDM, this amount of radiation dose reduction is possible.

## Discussion

Previous studies have shown promising results in comparing 2D synthesized images to the original FFDM^7,9,10^ and have shown that it can potentially be used in clinical practice. A study by Gur *et al*. on an earlier version of 2D synthesis found a slight decrease in cancer detection sensitivity, but with comparable specificity and no significant difference in recall rate^19^.

The patients included in our study are representative of the population that presents to the breast imaging unit in our centre. 54.7% of patients belong in “heterogeneously dense” and “extremely dense” categories, which is expected in an Asian population^14^.

In our study, we have shown that there is excellent agreement in all readers between C-View and FFDM in BI-RADS category and density assessment. When C-View is compared to FFDM, the sensitivity (94–97%) and specificity (98–100%) of C-View are consistently high in all readers. The PPV (88–100%) and NPV (99%) of C-View were also consistent and good. The positive predictive values (83–92%) were both high in FFDM and C-View with histopathology as the standard of reference.

Mass and calcification characterisation are comparable between C-View and FFDM. However, there are consistently more calcifications reported in C-View compared to FFDM in all readers (5–17% of cases). These are all of benign features and did not contribute towards a decision to biopsy, which translated to BI-RADS 2 (benign) category instead of BI-RADS 1 (negative) in clinical practice. As more calcifications were seen in C-View, a higher rate of recommendation for closer follow up may occur as compared to viewing them on original FFDM images alone. Slightly more architectural distortion was also reported in C-View in 2 readers (1% cases). These findings correlate to previous studies which noted that in some cases the appearance of microcalcifications, are better seen in C-View compared to FFDM, with a more conspicuous appearance of architectural distortions and spiculations^20^.

Radiation dose is one of the biggest factor that drives the industry and academic institute alike in using the synthesized 2D images instead of FFDM in clinical practice. The current practice of Combo mode exposes a patient twice for FFDM as well as tomosynthesis, hence almost doubling the radiation dose. Our study has shown that there is 52% reduction in radiation dose when C-View is used and only tomosynthesis images are acquired. Previous studies by Zuckerman *et al*. and Skaane *et al*. have shown 39% and 45% reduction in radiation dose respectively with implementation of synthetic 2D images^7,9^.

Other measures to reduce radiation dose in DBT include using one-view tomosynthesis. A study by Genraro *et al*. on one-view-DBT using single view MLO tomosynthesis acquisition with single view CC FFDM showed no inferiority of the former to the standard 2 view FFDM^21^. A similar approach by Wallis *et al*. showed no difference in classification accuracy between 2D mammography and one-view tomosynthesis^22^. No differences were found between one-view tomosynthesis and dual-view FFDM or between one-view tomosynthesis and the combined modality (single view tomosynthesis with opposite FFDM view) by Svahn *et al*.^23^. However, there is substantially larger improvement in cancer detection with the use of two-view DBT^1,20,24^, hence the approach of synthesized 2D images and DBT is more appropriate for clinical practice.

Tomosynthesis allows less compression onto the breast during image acquisition as the problem with tissue overlapping was the limiting factor to do so in FFDM^20^. By obviating FFDM in clinical practice, the compression applied onto breasts during examination may be reduced. Furthermore, the examination time is also reduced, inadvertently shortening compression time and increasing patient comfort^20^.

Previous study by Upadhyay *et al*. have addressed the effect of addition of DBT to digital mammography on recall rates and reader confidence, however, it is not applicable in our study population as we do not have a population-based national screening program^25^. The screening program in Malaysia is of opportunistic and targeted nature^26^.

Studies have investigated the utilization of artificial intelligence and computer aided diagnosis in DBT and synthesized 2D images for breast lesions characterization^27^. Application of these methods into our study population is a consideration for future studies.

### Limitation

We acknowledge there are several limitations of our study. We only evaluated the use of synthesized 2D images in a single institution (UMMC), involving multi-ethnic Malaysian population with a single vendor (Hologic) and using a vendor specific software (C-View^TM^). Results from our study should be validated by other centres or in a multicentre study.

The data was collected since initial installation of the tomosynthesis machine in our unit and there have been several subsequent technical improvements, software upgrades and training. These results were also a reflection of the readers’ initial experience reading tomosynthesis and C-View images.

As our centre is not a dedicated screening program provider and there is no such structured program available in Malaysia, the number of patients and readers are limited in comparison to other published studies comparing the synthesized 2D images to FFDM.

## Conclusion

Our study has shown that synthesized 2D images (C-View) are comparable to 2D FFDM in lesion detection and characterisation, BI-RADS category assessment and density assessment when reviewed with DBT within the limitation of the study population. The sensitivity, specificity, PPV and NPV of synthesized 2D images are also comparable to FFDM. There is substantial reduction in radiation dose to the breasts when using synthesized 2D images instead of FFDM (up to 52%).

Hence, results from our study and recent publications contribute to providing more evidence that the synthesized 2D images are acceptable to be used in routine clinical practice, obviating the need for FFDM.

## Supplementary information

Video example of digital breast tomosynthesis

Image example of the 2D FFDM and synthesized 2D images (C-View)

## Data Availability

The datasets generated during and/or analysed in this study are available in the figshare repository, https://figshare.com/s/dd735e342f0fc83da7eb.
