# Supplementary material for: Diagnostic Efficacy of Synthesized 2D Digital Breast Tomosynthesis in Multi-ethnic Malaysian Population
Source: Sci Rep. 2019 Feb 6;9:1459. doi: 10.1038/s41598-018-37451-4 (PMC6365555; doi:10.1038/s41598-018-37451-4)
Supplement: Supplementary file 2 — Image example of the 2D FFDM and synthesized 2D images (C-View) [file 41598_2018_37451_MOESM2_ESM.docx]

Title: Supplementary information. Image example of Full field digital mammography and its synthesized 2D image

Manuscript title: **Diagnostic Efficacy of Synthesized 2D Digital Breast Tomosynthesis in Multi-ethnic Malaysian Population**

Authors: Ab Mumin N_,_ *Rahmat K, Fadzli F, Ramli M, Westerhout C, Ramli N, Rozalli F, Ng KH

*Correspondence to: katt_xr2000@yahoo.com


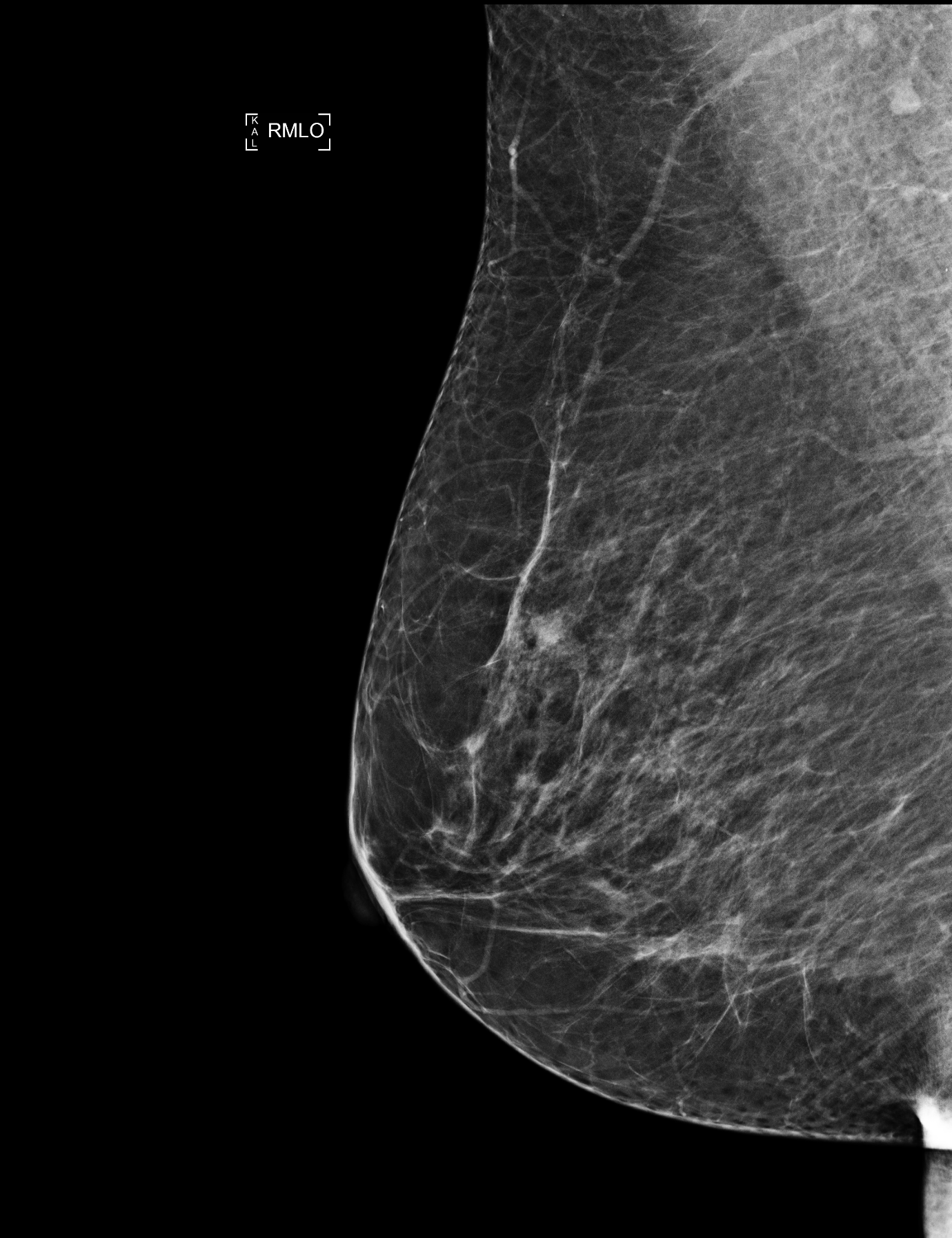


Figure1: example of 2D Full field digital mammography image (FFDM)


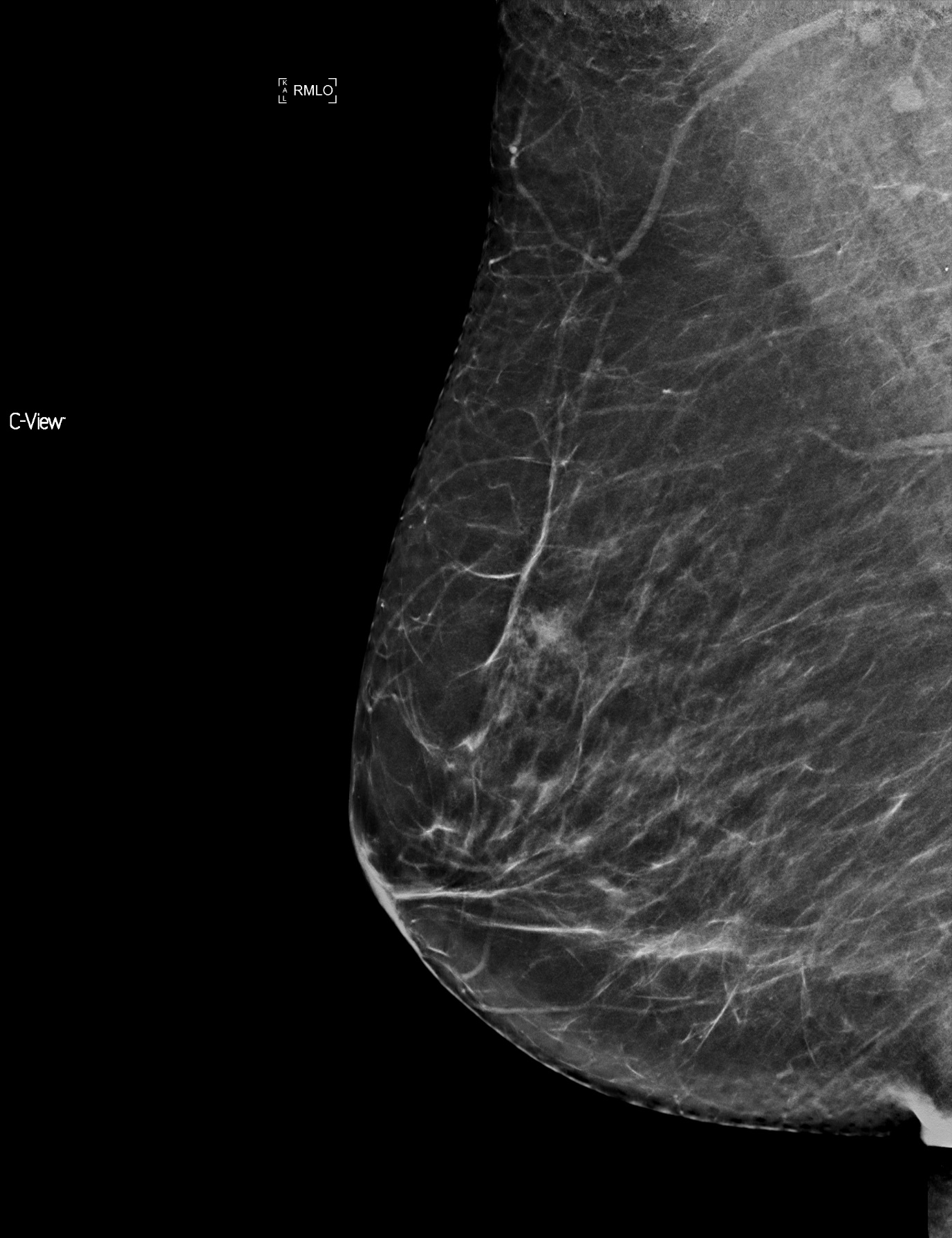


Figure 2: example image of C-View (2D synthesized image from tomosynthesis images)
